# Prevalence of Sexual Aggression Victimization and Perpetration in a German University Student Sample

**DOI:** 10.1007/s10508-021-01963-4

**Published:** 2021-06-30

**Authors:** Barbara Krahé, Isabell Schuster, Paulina Tomaszewska

**Affiliations:** Department of Psychology, University of Potsdam, Karl-Liebknecht-Str. 24-25, 14476 Potsdam, Germany

**Keywords:** Sexual aggression, Sexual victimization, Male victims, Female perpetrators, Same-sex relationships

## Abstract

This study examined the prevalence of sexual aggression perpetration and victimization in a sample of 1,172 students (755 female, 417 male) from four universities in Germany. All participants were asked about both victimization by, and perpetration of, sexual aggression since the age of 14 years, using the Sexual Aggression and Victimization Scale (SAV-S). Prevalence rates were established for different coercive strategies, sexual acts, and victim–perpetrator relationships. Both same-sex and opposite-sex victim–perpetrator constellations were examined. The overall victimization rate was 62.1% for women and 37.5% for men. The overall perpetration rate was 17.7% for men and 9.4% for women. Prevalence rates of both victimization and perpetration were higher for participants who had sexual contacts with both opposite-sex and same-sex partners than for participants with exclusively opposite-sex partners. Significant overlap was found between victim and perpetrator status for men and women as well as for participants with only opposite-sex and both opposite-sex and same-sex partners. A disparity between (higher) victimization and (lower) perpetration reports was found for both men and women, suggesting a general underreporting of perpetration rather than a gendered explanation in terms of social desirability or the perception of consent cues. The findings are placed in the international research literature on the prevalence of sexual aggression before and after the #metoo campaign, and their implications for prevention efforts are discussed.

**Supplementary Information:**

The online version contains supplementary material available at 10.1007/s10508-021-01963-4.

## Introduction

Sexual aggression has been recognized as a serious problem for young adults and has been studied extensively in college student samples (Fedina et al., 2018; Mellins et al., 2017). Sexual aggression describes a range of sexual activities, such as sexual intercourse, oral sex, kissing, and sexual touching, imposed on another person against her or his will. It involves a range of coercive strategies, such as threat or use of physical force, exploitation of the victim’s inability to resist, abuse of a position of power, or verbal pressure (Krahé, 2021). This definition is broader than legal definitions of sexual assault, because it includes any form of nonconsensual sexual contact irrespective of whether it is penalized by law. Sexual victimization has been established as a risk factor for a range of adverse consequences that undermine survivors’ psychological, physical, and sexual well-being (e.g., Basile et al., 2020; Dworkin et al., 2017; Kelley & Gidycz, 2017).

Despite the progress made in understanding the scale of sexual aggression in college student samples, several aspects have received limited attention in the past. First, most of the evidence has been accumulated in North America, limiting the understanding of the scale of sexual aggression in other countries. Second, the majority of studies have examined male sexual aggression against female victims, neglecting the prevalence of male victimization and female perpetration. Third, research has focused on heterosexual victim–perpetrator constellations, paying less attention to the prevalence of sexual aggression in individuals with exclusively same-sex or both opposite- and same-sex sexual contacts. Fourth, the separation of victim and perpetrator roles along gender lines has hampered the systematic analysis of the degree to which victimization experiences and perpetration overlap and has precluded the analysis of alternative explanations for the widely observed disparity between the rates of reported victimization and perpetration. The present study was designed to address these issues by presenting data from a sample of male and female university students in Germany, who differed in sexual experience background and were asked to report both victimization and perpetration.

### Prevalence of Sexual Aggression in University Student Samples

#### Victimization

Reviews of the North American research literature have estimated that about one in five women will experience sexual victimization during their time in college (Muehlenhard et al., 2017), and reviews comparing victimization rates for men and women concluded that women are more at risk of experiencing sexual aggression (Fedina et al., 2018; Oswalt et al., 2018). The most recent AAU Campus Climate Survey conducted in 2019 with more than 180,000 students from 33 higher education institutions found that 25.9% of undergraduate women reported having experienced nonconsensual sexual contact by force or inability to consent, with rates ranging from 14 to 32% across universities. In almost all cases, the perpetrators were male, and in the majority of cases, they were known to the victims as current or former partners or friends (Cantor et al., 2020). In the same survey, 6.8% of undergraduate men reported having experienced nonconsensual sexual contact by force or inability to consent, which is about a third of the female victimization rate (Cantor et al., 2020). About two-thirds of male victims reported that the perpetrator had been a woman. More similar rates of sexual victimization prior to entering college of 67% for women and 55% for men were found by Cusack et al. (2019). These figures include rates of 21% and 18%, respectively, for sexual assault involving force or threat of harm. The review by DePraetere et al. (2020) established that a third of the included 33 studies found higher victimization rates for men than for women.

There is consistent evidence that victimization rates vary depending on sexual orientation and sexual behavior and are elevated in individuals who identify as LGBTI or who have sex with both male and female partners. A survey by Martin et al. (2011) found that lesbian and bisexual women had a significantly higher rate of sexual victimization, both before and during their time in college, compared with heterosexual women. Another study found significantly elevated rates of sexual victimization among bisexual women and homosexual men (Mellins et al., 2017). Thus, differences in individuals’ sexual orientation and sexual behavior appear to be related to differences in the likelihood of experiencing sexual victimization.

#### Perpetration

With the exception of the study of convicted sex offenders, the research literature on self-reported perpetration of sexual aggression is much smaller than the victimization literature, and the same is true for prevention efforts, which also tend to neglect perpetrators (Mahoney et al., 2019). A review of 78 studies with more than 25,000 college men conducted between 2000 and 2017 found an average perpetration rate across different forms of sexual aggression of 29.3%, and an average rate of 6.5% for rape (Anderson, et al., 2019). The prevalence rates varied from 6.7 to 92.0% between studies, and part of this variation was attributable to differences in measurement tools. In a sample of male college students, 18% reported some form of sexual aggression perpetration since the age of 14 (Brennan et al., 2019), and a study with an online sample of college men found a prevalence rate of 35%, based on behaviorally specific items of the Sexual Experiences Survey (Pegram et al., 2018).

Even less evidence is available on female perpetrators of sexual aggression. Moreover, information about the rate of women among perpetrators of sexual aggression is often derived from reports of victims, who are asked about the gender of the perpetrator, rather than being based on perpetrator self-reports (see review by Stemple et al., 2017) or limited to the study of female sex offenders (see review by Fisher & Pina, 2013). The few studies that have collected self-reports of perpetration from women are based on small sample sizes (e.g., Bouffard & Goodson, 2017; Hughes et al., 2020). A study of sexual aggression toward intimate partners yielded a self-reported perpetration rate of 17% for women, compared with a rate of 27% for men (Brousseau et al., 2011). Overall, women’s sexual aggression perpetration tends to consist primarily in the use of verbal coercion or exploitation of the victim’s inability to resist, rather than the use or threat of physical force (e.g., Hughes et al., 2020).

#### Evidence from Germany

Most of the existing evidence on the prevalence of sexual aggression victimization and perpetration has been gathered in North America, with other parts of the world notably underrepresented (Dworkin et al., in press). For Germany in particular, evidence on the scale of young people’s experience of sexual victimization is scarce, and data on self-reported perpetration almost completely lacking. Although Germany is also a western country, the extent to which sexual aggression perpetration and victimization rates are comparable to findings from North America using a similar methodology is an empirical question. In Germany, students are generally older than American students when they start university (in 2019, the average age of first-year students was 21.7 years; Statistica, 2020), the majority of students live off campus, and differences in variables relevant to sexual aggression, such as drinking habits and dating patterns, may affect the odds of sexual aggression victimization and perpetration.

A representative survey of German adolescents and young adults between 14 and 25 years of age revealed that 20% of female and 4% of male respondents without a migration background reported having been pressured into nonconsensual sexual acts at least once (Bode & Heßling, 2015), with slightly higher rates for respondents with a migration background. A study with a representative sample of women aged 21–40 years found a lifetime prevalence rate of sexual victimization of 5.4%, based on responses to a single question about attempted or completed penetration through the use or threat of force (Hellmann et al., 2018). Another study with a representative sample of adults above the age of 18 years found rates of sexual victimization in the last 12 months of 1.2% for women and 0.6% for men (Allroggen et al., 2016). All three studies used broad questions about sexual victimization that did not specify different coercive strategies or sexual acts, which is considered problematic, as it is likely to lead to an underreporting of victimization experiences (Cook et al., 2011).

Collecting reports of both victimization and perpetration from all respondents and using behaviorally specific questions, a study with a convenience sample of more than 2,000 university students found an overall rate of victimization since the age of 14, the age of consent in Germany, of 35.9% for women and 19.4% for men (Krahé & Berger, 2013). The perpetration rate was 13.2% for men and 7.6% for women. The rates of both victimization and perpetration were highest among participants who had sexual contacts with both opposite-sex and same-sex partners, consistent with the findings from North American studies mentioned above. Sexual aggression and victimization rates were higher between current or former partners and acquaintances than between strangers, disconfirming the “real rape” stereotype (Krahé, 2016).

#### Victim–Perpetrator Overlap

Due to the traditional separation of victimization and perpetration studies along gender lines, the question of victim–perpetrator overlap has received little attention in past research. This question is relevant for understanding the dynamics of sexual assault experiences in individual biographies and for establishing adequate support and prevention measures (Peterson et al., 2019). Studies that collected both victimization and perpetration reports from the same participants have found consistent evidence that a substantial proportion of respondents reported both victimization experiences and perpetration behavior (e.g., Meadows et al., 2020; Ybarra et al., 2016). In a large sample of university students in Germany, 6.3% of women and 6.6% of men reported at least one form of sexual victimization and perpetration since the age of 14, compared with 29.2% of women and 12.4% of men who reported only victimization and 1.5% of women and 6.7% of men who reported only perpetration (Krahé & Berger, 2020). This study, as well as the study by Peterson et al. (2019), found significant associations between victimization and perpetration reports for men and women. The present study was designed to test the replicability of this finding using the same methodology as the study by Krahé and Berger (2020).

#### Reporting Disparity

Collecting both victimization and perpetration reports from the same participants is also required for testing alternative explanations for the widely found disparity between (higher) victimization and (lower) perpetration reports (Kolivas & Gross, 2007). A study on youth sexual aggression by Ybarra et al. (2016) found that a victimization rate for females of 13.6% contrasted with a male perpetration rate of 4.6%, and a male victimization rate of 8.3% contrasted with a female perpetration rate of 1.6%. However, in the majority of research discussing the reporting disparity, victimization reports were only available from females and perpetration reports only from males, which means that sex of participant is confounded with victim versus perpetrator role. In the present study, this confound was avoided by comparing the gap between women’s victimization and men’s perpetration reports with the gap between men’s victimization and women’s perpetration reports among participants with opposite-sex contacts only.

Competing explanations have been offered for the disparity between victimization and perpetration reports (Kolivas & Gross, 2007). One explanation refers to the difference in point of view underlying victim and perpetrator reports. Whereas victims have first-hand knowledge about whether or not they were made to engage in sexual acts against their will, perpetrators need to infer the nonconsensual nature of the sexual activity from the victim’s response to their sexual advances. According to this explanation, perpetration reports could be lower because perpetrators fail to recognize the sexually aggressive character of their behavior toward the victim, and it should hold for male and female perpetrators in the same way. A second interpretation refers to sex differences in the interpretation of sexual interest cues. There is a wide literature documenting men’s misperception of women’s friendliness cues as sexual interest (e.g., Abbey et al., 1998; Lee et al., 2020), suggesting that men fail to recognize women’s nonconsent in response to their sexual advances. By this reasoning, men should be more likely than women to underreport perpetration, resulting in a larger disparity between victimization and perpetration reports for men than for women. A third explanation suggests that participants might be less willing to report perpetration than to report victimization due to the socially undesirable character of sexually aggressive behavior. Based on this reasoning, one would expect the disparity to be larger for women than for men, because aggressive behavior in general, and sexual aggression in particular, is even less acceptable for women than for men (see Krahé, 2021, for gender differences in aggression). In the present study, these explanations could be tested against each other.

### The Current Study

Based on the state of knowledge summarized above, our study examined the prevalence of sexual aggression victimization and perpetration since the age of 14 years in a sample of students from four universities in the states of Brandenburg and Berlin in Germany. Building on an earlier study by Krahé and Berger (2013), we sought to identify the rates of different forms of sexual aggression perpetration and victimization and analyze them in relation to gender differences and sexual experience in opposite-sex and/or same-sex contacts. Reports of perpetration and victimization were broken down by prior victim–perpetrator relationship, distinguishing between (a) current or former partners, (b) friends or acquaintances, and (c) strangers. The design of the study enabled us to address the overlap between victim and perpetrator roles found in past research, which has implications for both primary and secondary intervention measures and to examine alternative explanations for the widely found disparity between women’s victimization reports and men’s perpetration reports.

The following predictions were examined: (1) Prevalence rates for sexual victimization are higher for women than for men (Hypothesis 1). (2) Prevalence rates for sexual aggression perpetration are higher for men than for women (Hypothesis 2). (3) Individuals who engage in both opposite-sex and same-sex sexual contacts have higher rates of both perpetration and victimization than do individuals who engage in opposite-sex or same-sex contacts only (Hypothesis 3). (4) There is a significant overlap between reports of victimization and perpetration (Hypothesis 4). (5) A reporting disparity in the form of higher victimization reports by one gender and lower perpetration reports by the other gender will be found for both men and women (Hypothesis 5).

## Method

### Participants

A total of 1,172 undergraduate students (755 women, 417 men) participated in the study. The data were collected as the baseline measure of an intervention study designed to reduce the risk of sexual aggression perpetration and vulnerability of sexual victimization. Participants were recruited from four universities in the federal states of Brandenburg and Berlin in Germany. The mean age of the sample was 22.59 years (SD 3.51, range 18–35), and 94% were German nationals. On average, participants were in their second year at university, *M*_*semesters*_ = 3.74 (SD 3.12; median = 3). Almost all participants (97.2%) had experience of sexual intercourse, similar to a representative sample of young adults in Germany, where the rate for the 23-year-olds was 93% (Bode & Heßling, 2015). The age at first sexual intercourse was 16.77 years (SD 2.20) and did not differ between male (*M* = 16.3 years) and female (*M* = 16.74 years) participants. In terms of sexual experience background, 72.1% reported exclusively opposite-sex contact, 3.5% reported exclusively same-sex contact, and 24.3% reported both opposite- and same-sex contact. A small minority of 6% (37 women and 34 men) reported neither opposite-sex nor same-sex contact. The majority of participants (88.8%) were in a steady relationship at the time of the survey or had been in a steady relationship in the past, with no significant gender difference on this variable. Detailed information about participants’ sexual orientation, sexual experience background and number of sexual partners is presented in the Supplementary Material.

### Measures

#### Sexual Aggression Victimization and Perpetration 

The Sexual Aggression and Victimization Scale (SAV-S; Krahé & Berger, 2013) was used to assess the prevalence of sexual aggression victimization and perpetration since the age of 14, the age of consent in Germany. Building on Koss et al.'s (2007) revised version of the SES, the SAV-S differentiates between three coercive strategies (threat or use of physical force, exploitation of the inability of the victim to resist, and use of verbal pressure) and four sexual activities (sexual touch, attempted penetration, completed penetration, and other sexual acts, e.g., oral sex). In addition, the SAV-S breaks down the reports by three different relationship constellations between victim and perpetrator (current/former partner, friend/acquaintance, and stranger). Altogether, the SAV-S comprises 36 items each for victimization and perpetration (three coercive strategies × three victim–perpetrator constellations × four sexual acts). Parallel questions are asked about victimization and perpetration. An example of the question format is presented in the Supplementary Material (Table SM1). The victimization items are always presented first to give victims the opportunity to report their victimization experiences before being confronted with the perpetration items. The instrument was validated in a qualitative study by Krahé et al. (2016) and has been used in a wide range of countries (Krahé et al., 2015; Schuster et al., 2016a, 2016b; Tomaszewska & Krahé, 2018).

Participants received a version tailored to their gender and sexual experience background. Three screening questions were used: (1) gender (female/male/other), (2), whether they ever had sex with a man, and (3) whether they ever had sex with a woman. For example, women who reported only opposite-sex contacts received questions referring to a male perpetrator (victimization part) and a male victim (perpetration part). Women who reported only same-sex contacts received questions referring to a female perpetrator (victimization part) and a female victim (perpetration part). This combination yielded a total of nine versions. Participants who reported no sexual contact with either opposite-sex or same-sex partners were assigned to the victimization version for individuals with both opposite-sex and same-sex contacts. The response format was *no* (0) or *yes* (1) for the opposite-sex contact only and same-sex contact only questionnaire versions, and *no* (0), *yes, a man* (1), and *yes, a woman* (2) for both opposite-sex and same-sex contact questionnaire versions. “Yes” responses in that version were collapsed into a dichotomous score (no/yes).

#### Demographic, Relationship, and Sexual Experience Information

Participants were asked to indicate their gender, age, university, study semester, subject of study, nationality, sexual orientation, relationship experience and sexual experience background (whether they ever had sexual contact with a man, a woman, or both).

### Procedure and Data Cleaning

The study was advertised as a study on young adults’ competence in sexual situations. Participants were informed that the study was designed to examine ways for promoting the appraisal of sexual situations, to communicate one’s desires and boundaries in a clear fashion, and to recognize the partner’s desires and boundaries. It was mentioned that the topic of nonconsensual sex would be also included in the study. Invitations to participate in the study were sent out to students enrolled in four higher education institutions in the Brandenburg and Berlin region through the respective student offices or student associations. Students interested in participating registered in a data bank created for the purposes of this study. Of the 1,499 students who registered their interest in the data bank, 66 cases had to be excluded because they provided no or incomplete email addresses for contact or registered more than once with the same email address. A total of 1,433 students received the link to the survey, of whom 1,238 completed it to the end, amounting to a response rate of 86.4%. Thirty data sets were excluded because they were suspicious (e.g., had identical personalized codes, age, gender, and other demographic information). Fourteen participants were excluded because they had extremely short or long completion times, as identified by z-transformed scores (Field, 2018). A further 15 participants were excluded because they were above the age cut-off of 35 years or did not indicate their age. Seven participants were excluded because they indicated “other” as their gender, and that group was too small to warrant separate analysis. This low frequency was expected in an unselected student sample. However, according to the aim of the SAV-S to provide a gender-inclusive assessment tool, the third gender category was included as an option. This elimination process led to the final sample of 1,172 included in this study.

All materials were presented in German in an online format. Participants were required to give active consent on the first page of the survey before being able to proceed to the items. They were informed that they could terminate the survey at any point. At the end of each page of the SAV-S, a link to a list of counselling agencies for victims and perpetrators of sexual aggression could be accessed via a “help” button. All participants received an Amazon voucher for their participation. A separate browser tab was opened at the end of the survey for participants to leave their contact email address, so that the address for sending the voucher was stored separately from the survey responses.

### Plan of Analysis

The analyses were conducted in five steps. First, overall prevalence rates based on all participants who endorsed at least one of the victimization or perpetration items were calculated for men and women and for participants in each gender group differing in sexual experience background. Because of the low frequencies of women and men in the group with only same-sex contact, comparisons by sexual experience background were conducted only for the groups of participants with opposite-sex only and both opposite- and same-sex contacts in this and all following steps. Second, frequencies were calculated for each item of the victimization and perpetration measures. Third, an ordinal score in which participants were categorized by the most serious form of sexual aggression victimization and perpetration reported was created, based on previous research (Johnson et al., 2017; Koss et al., 2007, 2008). Fourth, the association between victim and perpetrator reports was calculated based on the number of participants who reported both victimization and perpetration. Significance tests for comparisons between gender groups and groups differing in sexual experience background were conducted only for comparisons involving cells with more than 20 cases, following the guideline by Black et al. (2011). For contingency tables with *df* = 1, *χ*^2^ values were converted into Cohen’s *d* or odds ratios as a measure of effect size, based on https://www.psychometrica.de/effect_size.html#transform. Significant omnibus *χ*^2^ tests were followed up by post-hoc tests based on the adjusted cell residuals (Sharpe, 2015). Fifth, we examined the disparity in victimization and perpetration reports for male and female participants by calculating perpetration reports in proportion to victimization reports.

## Results

### Overall Rates of Sexual Aggression Victimization and Perpetration

The overall rate of victimization was 62.1% for women and 37.5% for men, as shown in Table 1. The gender difference was significant, χ^2^(1, 1152) = 63.80, *p* < .001, *d* = 0.48, consistent with Hypothesis 1. The overall rate of perpetration was significantly higher for men (17.7%) than for women (9.4%), χ^2^(1, 1154) = 16.90, *p* < .001, *d* = 0.24 (see Table 1). This pattern is consistent with Hypothesis 2. Also shown in Table 1, victimization rates for participants with both opposite-sex and same-sex contacts were significantly higher than for participants with exclusively opposite-sex contacts in both gender groups, women: χ^2^(1, 700) = 8.10, *p* = .004, *d* = 0.22; men: χ^2^(1, 345) = 7.58, *p* = .006, *d* = 0.30. These findings are consistent with Hypothesis 3. Men and women who had sex with both members of the opposite and the same sex also had the highest prevalence of sexual aggression perpetration, but the difference with the opposite-sex-only group was nonsignificant.

**Table 1:** Overall prevalence of sexual aggression and victimization for men and women by sexual experience background

	Victimization %			Perpetration %			
	Women	Men	χ^2^^a^	Women		Men	χ^2^^a^
*All*	*62.1* (464/747)	*37.5* (152/405)	63.80***	*9.4* (70/747)		*17.7* (72/407)	16.90***
Opposite-sex partners only	60.8 (305/502)	32.7 (91/278)	56.22***	9.4 (47/501)		16.7 (47/282)	9.07**
Opposite-and same-sex	72.2 (143/198)	50.7 (34/67)	10.41**	11.2 (22/197)		22.4 (15/67)	5.22
χ^2b^	8.10**	7.58**		0.51		1.21	

^a^Denotes differences between gender groups; ^b^Denotes differences between sexual experience groups within each gender. Corrected alpha level: .05/4 = .0125. *** *p* < .001; ** *p* < .0125. In parentheses: *n* victims/*n* total group size

### Item-Level Frequency Counts of Victimization and Perpetration

To yield a more fine-grained picture of the forms of sexual aggression experienced and committed, the frequency of “yes” responses was counted for each item, as presented in Table 2. For women, victimization rates across victim–perpetrator relationships and sexual acts were highest for the items referring to the use or threat of physical force (45.5%), followed by the exploitation of the inability to resist (38.9%), and the use of verbal pressure (28.9%). Across coercive strategies and sexual acts, rates were highest for sexual victimization by strangers (41.5%), followed by sexual victimization by friends or acquaintances (34.0%), and current or former partners (29.8%). For men, victimization rates across victim–perpetrator relationships and sexual acts were highest for the exploitation of the inability to resist (21.2%), followed by the use or threat of physical force (20.8%), and the use of verbal pressure (17.0%). Across coercive strategies and sexual acts, rates were highest for sexual victimization by strangers (20.6%), followed by friends or acquaintances (18.7%), and current or former partners (17.6%). In all of these comparisons, the rates for women were significantly higher than those for men, consistent with Hypothesis 1. Only few of the individual items could be tested for significant gender differences based on the criterion of a minimum cell size > 20, but all significant comparisons were in the direction of higher rates for women than for men.

**Table 2:** Sexual victimization in percent by gender, coercive strategy, relationship constellation, and type of sexual act since age 14

Victim—perpetrator relationship	Sexual activity	Coercive strategy (%, *n* in parentheses)							
		Use/threat of physical force		Exploitation of inability to resist		Verbal pressure		Overall (at least one yes response per row)	
		Women	Men	Women	Men	Women	Men	Women	Men
(Ex-)partner	Touching	**14.4 (109)**	6.5 (27)**	**9.9 (75)**	**6.0 (25)**	**13.7 (103)**	**8.3 (34)**	**24.4 (183)**	14.4 (59)**
	Attempt. penetration	9.3 (70)	3.6 (15)	6.6 (50)	4.1 (17)	**11.9 (89)**	**5.8 (24)**	**17.5 (131)**	9.5 (39)**
	Compl. penetration	5.9 (44)	1.7 (7)	4.8 (38)	2.2 (9)	**9.4 (71)**	**6.1 (25)**	**13.3 (99)**	7.6 (31)*
	Other (e.g., oral sex)	5.7 (43)	2.4 (10)	3.9 (29)	1.9 (8)	8.4 (63)	3.9 (16)	**12.2 (91)**	5.9 (24)**
Total (Ex-)partner		***17.4 (131)***	7.1 (29)**	***11.9 (89)***	***6.8 (28)***	***18.4 (138)***	***12.1 (50)***	***29.8 (222)***	17.6 (71)**
Friend/Acquaintance	Touching	**18.9 (142)**	9.3 (39)**	**18.2 (137)**	7.5 (31)**	**11.3 (85)**	**6.0 (25)**	**31.1 (233)**	16.1 (67)**
	Attempt. penetration	7.2 (54)	4.0 (17)	8.2 (62)	2.7 (11)	6.1 (46)	3.9 (16)	**14.1 (105)**	7.2 (30)**
	Compl. penetration	3.7 (28)	1.9 (8)	5.2 (39)	2.67(11)	3.9 (29)	2.2 (9)	8.8 (65)	4.4 (18)
	Other (e.g., oral sex)	5.3 (40)	2.4 (10)	5.7 (43)	2.2 (9)	5.3 (40)	2.4 (10)	**11.0 (82)**	4.9 (20)**
Total friend/acquaintance		***20.7 (156)***	11.0 (46)**	***20.1 (151)***	9.2 (39)**	***12.5 (94)***	***7.3 (30)***	34.0 (254)*	18.7 (77)*
Stranger	Touching	**30.7 (231)**	10.6 (45)**	**25.1 (188)**	12.3 (51)**	8.4 (63)	3.3 (14)	**39.4 (295)**	18.8 (78)**
	Attempt. penetration	6.3 (47)	2.6 (11)	6.0 (45)	1.4 (6)	3.5 (26)	1.9 (8)	9.5 (70)	4.1 (17)
	Compl. penetration	2.1 (16)	1.2 (5)	2.5 (19)	1.2 (5)	0.9 (7)	1.2 (5)	3.7 (27)	2.7 (11)
	Other (e.g., oral sex)	3.4 (26)	2.4 (10)	4.0 (30)	2.1 (9)	2.0 (15)	1.2 (5)	5.4 (40)	4.6 (19)
Total stranger		***32.8 (247)***	12.3 (51)**	***26.6 (200)***	13.3 (55)**	*8.9 (67)*	*3.9 (18)*	***41.5 (311)***	20.6 (85)**
Total coercive strategy		***45.5 (342)***	20.8 (85)**	***38.9 (291)***	21.2 (87)**	***28.9 (217)***	17.0 (70)**	***62.1 (464)***	37.5 (152)**

Multiple responses were possible. Figures in bold denote cell sizes with frequencies > 20 for which *χ*^2^ tests for significant gender differences were conducted, based on a corrected significance level of .05/33 tests = critical *p* < .002. * *p* < .002; ** *p* < .001

For perpetration, prevalence rates were much lower and displayed a somewhat different gendered pattern, as shown in Table 3. For women, the perpetration rate across victim–perpetrator relationships and sexual acts was highest for the items referring to verbal pressure (4.4%), followed by the exploitation of the inability to resist (3.5%), and the use or threat of physical force (3.2%). Across coercive strategies and sexual acts, rates were highest for sexual aggression against a current or former partner (6.4%), followed by sexual aggression against a friend or acquaintance (3.7%), and strangers (1.3%). For men, perpetration rates across victim–perpetrator relationships and sexual acts were highest for the exploitation of the inability to resist (10.4%), followed by the use of verbal pressure (6.1%), and the use of threat of physical force (5.6%). Across coercive strategies and sexual acts, rates were highest for sexual aggression against a former or current partner (9.8%), followed by sexual aggression against a friend or acquaintance (9.2%), and a stranger (7.5%). Only eight categories had sufficiently high frequencies to test for significant gender differences in perpetration, and four of the comparisons yielded significant gender differences, all in the direction of higher rates for men than for women (unwanted sexual touch by a friend or acquaintance, overall perpetration against a friend or acquaintance, overall use of exploiting the other person’s inability to resist as a coercive strategy, and total perpetration across all items). The prevalence of sexual touch and total sexual aggression against a current or former partner across coercive strategies and of the use or threat of physical force and the use of verbal pressure across relationships and sexual acts did not differ significantly between men and women.

**Table 3:** Sexual aggression perpetration in percent by gender, coercive strategy, relationship constellation, and type of sexual act since age 14

Victim—perpetrator	Sexual activity	Coercive strategy (%, *n* in parentheses)							
		Use/threat of physical force		Exploitation of inability to resist		Verbal pressure		Overall (at least one yes response per row)	
Relationship		Women	Men	Women	Men	Women	Men	Women	Men
(Ex-)partner	Touching	1.9 (14)	3.1 (13)	1.7 (13)	3.1 (13)	3.2 (24)	3.4 (14)	**5.7 (43)**	**8.3 (34)**
	Attempt. penetration	0.5 (4)	1.4 (6)	0.9 (7)	1.7 (7)	1.5 (11)	2.2 (9)	2.7 (20)	4.6 (19)
	Compl. penetration	0.1 (1)	1.0 (4)	0.3 (2)	0.5 (2)	0.9 (7)	1.2 (5)	1.1 (8)	2.7 (11)
	Other (e.g., oral sex)	0.1 (1)	0.7 (3)	0.1 (1)	0.5 (2)	0.8 (6)	2.2 (9)	0.9 (7)	2.9 (12)
Total (ex-)partner		*2.0 (15)*	*3.6 (15)*	*1.9 (14)*	*3.6 (15)*	*3.7 (28)*	*4.6 (19)*	***6.4 (48)***	***9.8 (40)***
Friend/Acquaintance	Touching	1.1 (8)	2.7 (11)	1.9 (14)	4.1 (17)	0.9 (6)	1.7 (7)	**3.3 (25)**	7.7 (32)*
	Attempt. penetration	0 (0)	0.7 (3)	0.4 (3)	1.7 (7)	0.4 (3)	1.0 (4)	0.7 (5)	3.1 (13)
	Compl. penetration	0.1 (1)	0.5 (2)	0.3 (2)	0.7 (3)	0.3 (2)	0.2 (1)	0.5 (4)	1.2 (5)
	Other (e.g., oral sex)	0 (0)	0.5 (2)	0 (0)	1.2 (5)	0 (0)	0.7 (3)	0 (0)	2.4 (10)
Total friend/acquaintance		*1.2 (9)*	*2.9 (12)*	*2.0 (15)*	*5.3 (22)*	*1.1 (8)*	*2.6 (11)*	***3.7 (28)***	9.2 (38)**
Stranger	Touching	0.3 (2)	1.4 (6)	0.9 (7)	4.6 (19)	0.3 (2)	1.4 (6)	1.1 (8)	6.8 (28)
	Attempt. penetration	0.1 (1)	0.7 (3)	0 (0)	0.7 (3)	0 (0)	0.7 (3)	0.1 (1)	1.7 (7)
	Compl. penetration	0 (0)	0.5 (2)	0 (0)	0.5 (2)	0.1 (1)	0.5 (2)	0.1 (1)	1.0 (4)
	Other (e.g., oral sex)	0.1 (1)	0.2 (1)	0.1 (1)	0.2 (1)	0 (0)	0.5 (2)	0.1 (1)	1.0 (4)
Total stranger		*0.4 (3)*	*1.9 (8)*	*0.9 (7)*	*5.3 (22)*	*0.4 (3)*	*1.7 (7)*	*1.3 (10)*	*7.5 (31)*
Total coercive strategy		***3.2 (24)***	***5.6 (23)***	***3.5 (26)***	10.4 (43)**	***4.4 (33)***	***6.1 (25)***	***9.4 (70)***	17.7 (72)**

Multiple responses were possible. Figures in bold denote cell sizes with frequencies > 20 for which *χ*^2^ tests for significant gender differences were conducted, based on a corrected significance level of .05/7 tests = critical *p* < .007. * *p* < .007; ** *p* < .001

### Ordinal Score of Victim and Perpetrator Status

In a third step, we calculated nonredundant ordinal scores, classifying participants according to their most severe form of sexual victimization and sexual aggression perpetration reported, following previous research (Johnson et al., 2017; Koss et al., 2007, 2008). Five levels were distinguished: (0) No victimization/perpetration (“no” responses to all individual items); (1) Sexual contact without penetration (i.e., sexual touch) or other sexual acts, but no attempted coercion, coercion, attempted rape, and rape; (2) Sexual coercion, i.e., attempted or completed vaginal or anal penetration or other sexual acts using verbal pressure, but no attempted or completed rape; (3) Attempted rape, i.e., attempted vaginal, or anal penetration through exploitation of the victim’s inability to resist or threat or use of physical force, but no completed rape; and (4) Completed rape, i.e., completed vaginal or anal penetration through exploitation of the victim’s inability to resist or threat or use of physical force.

The rates of victimization and perpetration at each level of severity for women and men are presented in Table 4. A significant sex difference was found for the distribution of victimization reports as a whole, χ^2^(4, 1152) = 66.18, *p* < .001. Victimization rates at all levels of severity except sexual coercion were significantly higher for women than for men. The most serious form of sexual victimization, completed rape, was reported by 14.9% of women and 6.9% of men. Regarding perpetration, only the “no perpetration” and “sexual contact” categories had frequencies > 20 and could be compared statistically between men and women. Post-hoc *χ*^2^ tests showed that significantly more women than men were in the “no perpetration” category. Men were significantly overrepresented as perpetrators in the category of nonconsensual sexual contact. Proportionately more men than women were in the other three perpetration categories, but the numbers were too small to test for significant differences.

**Table 4:** Most severe form of sexual victimization and aggression (n in parentheses)

	Victimization %		Perpetration %	
	Women	Men	Men	Women
No victimization/perpetration	37.9 (283)	62.5 (253)*	82.3 (335)	90.6 (677)*
Sexual contact	28.2 (211)	19.3 (78)*	10.3 (42)	5.5 (41)*
Sexual coercion	7.1 (53)	4.9 (20)	2.2 (9)	1.7 (13)
Attempted rape	11.9 (89)	6.4 (26)*	2.7 (11)	1.5 (11)
Rape	14.9 (111)	6.9 (28)*	2.5 (10)	0.7 (5)

To correct for multiple testing, a corrected level of *p* = .05/8 = .006 was adopted for the post hoc tests comparing gender differences within the four victimization levels with frequencies > 20. Due to low cell frequencies, no post-hoc significance tests were conducted for perpetration reports in the sexual coercion, attempted rape, and rape categories, with a corrected level of *p* = .05/4 = .0125 for the remaining two levels. * = significant gender difference

### Victim–Perpetrator Overlap

The fourth step in the analysis examined the overlap between victimization and perpetration experience, comparing men and women and participants with exclusively opposite-sex contacts and both opposite-sex and same-sex contacts. In both gender groups and for participants with only opposite-sex contacts and both opposite- and same-sex contacts, there was a significant overlap between victim and perpetrator status, φ = 0.24, *p* < .001, for women; φ = 0.33; *p* < .001, for men; φ = 0.25, *p* < .001, for participants with exclusively opposite-sex contacts; and φ = 0.14, *p* < .05, for participants with both opposite-sex and same-sex contacts. Among perpetrators, 98.6% of women and 71.8% of men reported victimization experiences, compared with a victimization rate of 58.4% of women and 30.1% of men in the “no perpetration” category. Similarly, 84.9% of perpetrators in the group with opposite-sex contacts only and 83.3% of perpetrators in the group with both opposite-sex and same-sex contacts reported victimization, compared with 46.2% and 64.7% of nonperpetrators, respectively. The difference in victimization rates between perpetrators and nonperpetrators was significant in both gender groups, χ^2^(1, 739) = 43.42, *p* < .001, OR *2.48*, for women; χ^2^(1, 397) = 43.39, *p* < .001, OR 3.56, for men, and for both sexual-experience groups, χ^2^(1, 771) = 49.21, *p* < .001, OR 2.72, for the opposite-sex-only group, and χ^2^ (1, 268) = 5.25, *p* = .022, OR 1.67, for participants with both opposite-sex and same-sex sexual experiences. These findings are consistent with Hypothesis 4.

### Disparity between Victimization and Perpetration Reports

The final analysis addressed the disparity between (higher) victimization and (lower) perpetration reports. We compared (a) female victimization reports and male perpetration reports, and (b) male victimization reports and female perpetration reports. Only participants with exclusively opposite-sex contacts were included in this analysis. As shown in Table 1, 62.1% of women reported at least one experience of sexual victimization, whereas 17.7% of men reported at least one act of sexual aggression perpetration. Thus, the perpetration rate was 28.5% of the victimization rate, which indicates a disparity of 71.5%. The rate of male victimization of 37.5% contrasts with a rate of female perpetration of 9.4%, which represents a proportion of 25.1% that is equivalent to a disparity of 74.9%. Thus, in line with Hypothesis 5, the disparity was similar in both gender groups, speaking against an explanation of the victimization-perpetration disparity based on gender differences in the perception of sexual intent cues or the tendency to underreport perpetration due to social desirability concerns.

## Discussion

This study was designed to provide evidence from a sample of university students in Germany on the prevalence of sexual aggression victimization and perpetration since the age of 14, the legal age of consent. Systematic research on the scale of sexual aggression remains scarce in Germany, with no more than three studies since 2010 identified for a recent review of the international literature (Dworkin et al., in press). We collected both victimization and perpetration reports from female and male participants differing in sexual experience background. In addition to providing overall prevalence rates, reports were broken down by coercive strategies, victim–perpetrator relationships, and sexual acts to yield a more fine-grained picture of the different forms of sexual aggression victimization and perpetration. Moreover, we examined the overlap of victim and perpetrator status and tested competing explanations for the widely observed discrepancy between the magnitude of victimization and perpetration rates.

Because the present study used the same instrument as the earlier study by Krahé and Berger (2013), the findings can be directly compared. Substantially higher prevalence rates for victimization were found in the current than in the former study (62.1% vs. 35.9% for women and 37.5% vs. 19.4% for men). Rates of perpetration were also higher in the present than in the former study (17.7% vs. 13.2% for men and 9.4% vs. 7.6% for women), but the difference was much smaller. Because the present study was advertised as a study on competence in sexual situations, whereas the former study was advertised as a study on young adults’ nonconsensual sexual experiences, it is unlikely that victims may have self-selected into the sample to a greater extent in the present than in the former study. A possible explanation of the higher rates could be that the current study was conducted after the #MeToo campaign, which started in 2017. The campaign has created a heightened public awareness of the problem of sexual victimization and promoted the understanding that nonconsensual sexual activity is not limited to sexual intercourse through the use or threat of force. By broadening the definition of sexual assault, the movement may have reduced the tendency by victims to label only those nonconsensual experiences as sexual assault that involve the use of force and conform to the real-rape stereotype of a stranger attack in a dark alleyway (Donde et al., 2018). As a result, participants in the current study may have identified more experiences as nonconsensual compared with participants of the earlier study. The finding that rates were especially elevated for women’s reports of sexual touch through the use or threat of force by a stranger is consistent with this reasoning. The current findings are also higher than in many studies conducted in the U.S., but again, those studies were conducted prior to the #MeToo campaign. However, more studies are needed that use the same methodology to collect prevalence data and examine differences in the understanding of sexual assault pre- and post the start of #MeToo to corroborate this explanation.

Moreover, the majority of U.S. studies have used the Sexual Experiences Survey (Koss et al., 2007), and the review by Anderson et al. (2019) found that the average prevalence rate of self-reported perpetration was higher in studies using other instruments compared with SES-based studies (41% vs. 26%). Our study adopted a more fine-grained differentiation of victim–perpetrator relationships than the SES, which may have triggered more memories of nonconsensual experiences.

In line with previous studies, victimization rates were significantly higher for women than for men, and this was true for participants with exclusively opposite-sex contacts and those with both opposite- and same-sex contacts, as well as for three out of the four levels of the ordinal severity score of sexual victimization. Perpetration rates were significantly higher for men than for women in the total sample, but due to the smaller group sizes, the gender difference was not significant within the subgroups of participants with only opposite-sex contacts and with both opposite- and same-sex contacts. On the ordinal severity score, perpetration rates were significantly higher for men than for women for unwanted sexual contact, the only level for which cell frequencies were above 20. Moreover, the present results join a substantial body of evidence showing that individuals who engage in sexual contacts with both opposite- and same-sex partners report higher rates of sexual victimization compared with participants who only have opposite-sex contacts (Chen et al., 2020; Mellins et al., 2017). Also consistent with past research, prevalence rates of both victimization and perpetration were highest in the category of unwanted sexual contact (Fedina et al., 2018).

Because participants in the present study reported both victimization experiences and perpetration behavior, we could establish the degree of overlap between the two. In line with a previous study based on the SAV-S, significant associations between victimization and perpetration reports were found for both gender groups (Krahé & Berger, 2020), corroborating evidence based on male samples (Peterson et al., 2019) and extending it to participants with exclusively opposite-sex and both opposite- and same-sex contacts. This finding highlights the need to consider potential victimization experiences of perpetrators of sexual assault beyond recognizing the association of sexual assault perpetration with sexual abuse in childhood (Krahé & Berger, 2017a).

By eliciting self-reports of both victimization and perpetration from all participants, the present study could also address the disparity between perpetration and victimization reports found in the literature. In their critical analysis of the SES, Kolivas and Gross (2007) noted that women’s reports of victimization are consistently higher than men’s reports of perpetration. They discussed sex differences in the perception of sexual interest as a potential reason for men’s underreporting of sexual aggression. Despite differences in the overall level of victimization and perpetration, victimization reports in our study exceeded corresponding perpetration reports by about 3:1 for both men and women. The finding that the disparity is practically identical for men and women is inconsistent with an explanation in terms of a specific tendency of men to misperceive female sexual interest, as discussed by Kolivas and Gross (2007). It is also not supportive of an explanation based on social desirability concerns, which would suggest a greater disparity for women than for men, given that aggressive behavior is more normatively prohibited for women than for men (e.g., Lightdale & Prentice, 1994). Instead, at least three other explanations may be considered. The first is that both men and women underreport perpetration relative to victimization because they perpetrated against victims outside the present sample. To control for this explanation, matched reports of victimization and perpetration would be needed from couples. A recent study using such a design found that couples disagreed in 88% of cases of sexual victimization reported by one partner, but that there was no difference in disparity rates depending on the gender of the perpetrator (Kuijpers, 2020). Although the present data cannot rule out the problem that perpetration and victimization reports are not matched, they demonstrate that the disparity persists when ruling out this alternative explanation and is not moderated by gender. A second explanation is that men and women may underreport perpetration to the same extent, but for different reasons: men, because they misperceive women’s sexual interest, women, because they are aware that engaging in sexual aggression is a violation of female gender roles (Lee et al., 2020; Swan et al., 2018). Finally, it is compatible with an explanation based on differences in perspective, with victims having first-hand information about a lack of consent, whereas perpetrators need to infer nonconsent from the victims’ signals, especially for those forms of sexual aggression that do not involve physical force.

### Strengths and Limitations

We believe our study has several strengths. First, it provides data from a large sample of university students from Germany, facilitating comparisons with sexual assault research from the U.S. In addition to victimization reports, we collected reports of perpetration to create a better basis for interventions addressing perpetration, reflecting the statement by Mahoney et al. (2020, p. 585) that “changing the behavior of potential perpetrators is the only route to truly eliminating sexual assault.” Second, we used a comprehensive set of behaviorally specific items to break down victimization and perpetration reports by different coercive strategies, sexual acts, and victim–perpetrator relationships. Third, differences in prevalence rates of perpetration and victimization as a function of sexual experience background were examined, comparing participants who only had opposite-sex partners with participants who had both opposite-sex and same-sex partners. Fourth, the findings join earlier results in demonstrating that a substantial proportion of perpetrators were also victims of sexual aggression. Finally, the study provided a closer look at the disparity in reporting victimization and perpetration by disentangling the impact of victim vs. perpetrator perspective from gender differences.

At the same time, several limitations of the study must be noted. First, participants in this study represented a convenience sample of college students in two federal states in Germany. Although there is no reason to assume that the student body in the participating universities differed in a systematic way from the population of college students nationwide, the generalizability of the findings needs to be demonstrated in further research based on a representative sample of higher education institutions. Moreover, volunteer bias may have affected the prevalence rates in that participants with victimization experiences may have been more likely to opt into the study than participants without victimization experiences. This possibility, however, is not specific to the current study but applies to any study on sexual victimization, because even in studies that start off with representative samples, participants with victimization experiences may be more willing to volunteer to answer the questions.

Second, the group of participants who only had sexual contacts with same-sex partners was too small to warrant separate analysis. Third, the analysis of reporting disparities for victimization and perpetration was based on independent reports of victims and perpetrators rather than couple reports, so that underreporting of perpetration cannot be determined at the level of single incidents. The finding that both men and women underreported the perpetration of sexual coercion is in line with a study by Brousseau et al. (2011), who examined within-dyad disagreement on the occurrence of sexual coercion, also demonstrating the underreporting of perpetration. However, higher victimization compared to perpetration rates might be due to perpetrators who assault multiple victims, which could not be examined in the present study. Furthermore, because participants were asked about sexual aggression victimization and perpetration since the age of 14, we do not know what proportion of the reported incidents occurred after they had started university. Finally, the present cross-sectional data do not yield information about the consequences of victimization experiences on aspects of mental health and sexual well-being. However, a previous longitudinal study using the same instrument for measuring sexual victimization showed that the more serious participants’ victimization experiences, the more depressive symptoms they showed and the lower their sexual self-esteem was 1 year later (Krahé & Berger, 2017b).

Despite these limitations, the present findings contribute new data on the prevalence of sexual aggression victimization and perpetration in college student samples from Germany, where very little research on the issue has been conducted so far. The scale of the prevalence rates highlights the need for interventions designed to reduce perpetration and victimization rates. A broad range of evidence-based interventions has been designed and implemented in the U.S. (see DeGue et al., 2014; Orchowski et al., 2020, for reviews), but their applicability in other cultures has not been tested. In addition to approaches designed to promote bystander interventions in situations that carry the risk of a sexual assault (Kettrey & Marx, 2019), interventions should be developed that reduce the odds that such situations arise in the first place. First evidence collected in Germany about the modifiability of risk factors for perpetration and vulnerability factors for victimization has yielded promising results for designing theory-based intervention programs (Schuster et al., 2020). The present findings suggest that such programs are urgently needed.

## Supplementary Information

Below is the link to the electronic supplementary material.

Supplementary file1 (DOCX 22 KB)
