# Supplementary material for: Prevalence of Sexual Aggression Victimization and Perpetration in a German University Student Sample
Source: Arch Sex Behav. 2021 Jun 30;50(5):2109–21. doi: 10.1007/s10508-021-01963-4 (PMC8275504; doi:10.1007/s10508-021-01963-4)
Supplement: Supplementary file 1 — Supplementary file1 (DOCX 22 KB) [file 10508_2021_1963_MOESM1_ESM.docx]

Supplementary Material:

**Prevalence of Sexual Aggression Victimization and Perpetration**

**in a German University Student Sample**

**Detailed Information about Participants’ Sexual Orientation and Experience Background**

The majority of participants (82.5%) described their sexual orientation as heterosexual, 5.9% as homosexual, and 11.6% as bisexual. More women (14.3%) than men (6.9%) identified as bisexual, and more men (10.1%) than women (3.6%) identified as homosexual, χ^2^ (2,1134) = 31.04, *p* < .001. More men (7.2%) than women (1.7%) reported exclusively same-sex contacts, whereas more women (27.7%) than men (18.0%) reported both opposite-sex and same-sex contacts, χ^2^ (2,1101) = 30.70, *p* < .001.

The number of sexual partners in a steady relationship was *M* = 2.44 (*SD* = 1.87; Median = 2.00), and the mean number of casual sex partners was *M* = 6.46 (*SD* = 10.34; Median = 3.00). To handle outliers, scores were capped at + 3 *SD* above the mean. A MANOVA with gender and sexual experience (opposite-sex only, same-sex only, both) as between-subjects factors and number of steady and casual sex partners as dependent variables yielded significant multivariate main effects for gender, *F* (2, 914) = 12.68, *p* < .001, partial eta^2^ = .027, sexual experience, *F* (4, 1830) = 37.67, *p* < .001, partial eta^2^ = .076, and a significant multivariate interaction, F (4,1830) = 6.44, *p* < .001, partial eta^2^ = .014. All univariate effects except the gender effect on number of steady partners were significant. Women had fewer casual sex partners than men, *M*_Women_ = 5.9 (*SD* = 9.27); *M*_Men_ = 7.73 (*SD* = 11.99), *F* (1,921 = 20.51, *p* = .004. Participants with exclusively opposite-sex contacts had significantly fewer casual partners (*M* = 4.03, *SD* = 6.69) than did participants with exclusively same-sex (*M* = 12.29, *SD* = 14.91) and both same-sex and opposite-sex contacts (*M* = 12.09, *SD* = 14.29), *F* (2,921) = 62.40, *p* < .001. Participants with exclusively opposite-sex contacts had significantly more steady partners (*M* = 2.22, *SD* = 1.52) than participants with exclusively same-sex partners (*M* =1.26, *SD* =1.15), but fewer partners than participants with both opposite- and same-sex contacts (*M* = 3.56, *SD* = 2.48), *F* (2,921) = 31.97, *p* < .001. The interaction effects between sex and sexual experience were significant for both steady partners, *F* (2,921) = 5.44, *p* = .004, and casual partners, *F* (2,921) = 5.36, *p* = .005. They indicated that in the group with both opposite-sex and same-sex contacts, women had more steady partners than did men, *M*_Women_ = 3.74, *SD* = 2.52, *M*_Men_ = 2_._.81, *SD* = 1.93. With regard to casual partners, men in the same-sex only group reported more partners than did women, *M*_Men_ = 15.42, *SD* = 15.77, *M*_Women_ = 2_._13, *SD* = 2.36.

Table SM1

*Format of the SAV-S: Female Version, Victimization, Opposite-Sex Contacts Only*

1. Has a man ever made (or tried to make) you have sexual contact with him against your will by **threatening to use force or by harming you** (e.g., by hurting you, holding you down, or threatening to do so)?

| My **current or former partner** in a steady relationship to engage in ... | | | | |
| --- | --- | --- | --- | --- |
|  |  | yes | no |  |
| … sexual touch (kissing/petting) |  | Ο | Ο |  |
| … attempted intercourse |  | Ο | Ο |  |
| … completed intercourse |  | Ο | Ο |  |
| … other sexual acts (e.g., oral sex) |  | Ο | Ο |  |
| A **friend** or **acquaintance** to engage in... | | | | |
|  |  | yes | no |  |
| … sexual touch (kissing/petting) |  | Ο | Ο |  |
| … attempted intercourse |  | Ο | Ο |  |
| … completed intercourse |  | Ο | Ο |  |
| … other sexual acts (e.g., oral sex) |  | Ο | Ο |  |
| An **unknown man** (e.g., someone I met at a club) to engage in ... | | | | |
|  |  | yes | no |  |
| … sexual touch (kissing/petting) |  | Ο | Ο |  |
| … attempted intercourse |  | Ο | Ο |  |
| … completed intercourse |  | Ο | Ο |  |
| … other sexual acts (e.g., oral sex) |  | Ο | Ο |  |

1. Has a man ever made (or tried to make) you have sexual contact with him against your will **by exploiting the fact that you were unable to resist** (e.g., after you had had too much alcohol or drugs)?
2. Has a man ever made (or tried to make) you have sexual contact with him against your will **by putting verbal pressure on you** (e.g., by threatening to end the relationship or to spread lies)?
